# [Retracted] Epigenetic high regulation of ATAD2 regulates the Hh pathway in human hepatocellular carcinoma

**DOI:** 10.3892/ijo.2025.5837

**Published:** 2025-12-16

**Authors:** Gang Wu, Xiaojun Lu, Yawei Wang, Hui He, Xiangyu Meng, Shuguan Xia, Kunming Zhen, Yongfeng Liu

Int J Oncol 45: 351-361, 2014; DOI: 10.3892/ijo.2014.2416

Following the publication of the above article, a concerned reader drew to the Editor's attention that, regarding the immunohistochemical staining experiments shown in Fig. 2, the insets for the data panels 2A and B, and 2C and D, respectively were remarkably similar, such that these data may not have consistently been identifiable with the main images proper for these figure parts. Secondly, with the western blot data shown in Fig. 3c, the PTCH1/Huh7 and ATAD2/siRNA-HCCLM3 protein bands were remarkably similar, such that the same data were likely to have been duplicated in the figure where different experiments were intended to have been portrayed. Finally, the two sets of flow cytometric plots shown in Fig. 2A appeared to show similar groupings of dots, which would not have been anticipated if these experiments had been performed discretely under different experimental conditions, suggesting a fundamental flaw either in the way in which these experiments were performed, or in how the results were outputted.

After having conducted an internal investigation of the data in this paper, the Editor of *International Journal of Oncology* has decided that this article should be retracted from the journal on the grounds of an overall lack of confidence in the data. The authors were asked for an explanation to account for these concerns, but the Editorial Office did not receive a reply. The Editor sincerely apologizes to the readership for any inconvenience caused, and we thank the reader for bringing this matter to our attention.

